# Evidence from simulation studies for selective constraints on the codon usage of the Angiosperm *psbA* gene

**DOI:** 10.1371/journal.pcbi.1009535

**Published:** 2021-10-26

**Authors:** Antonina Kalkus, Joy Barrett, Theyjasvi Ashok, Brian R. Morton

**Affiliations:** Department of Biology, Barnard College, Columbia University, New York, New York, United States of America; Temple University, UNITED STATES

## Abstract

The codon usage of the Angiosperm *psbA* gene is atypical for flowering plant chloroplast genes but similar to the codon usage observed in highly expressed plastid genes from some other Plantae, particularly Chlorobionta, lineages. The pattern of codon bias in these genes is suggestive of selection for a set of translationally optimal codons but the degree of bias towards these optimal codons is much weaker in the flowering plant *psbA* gene than in high expression plastid genes from lineages such as certain green algal groups. Two scenarios have been proposed to explain these observations. One is that the flowering plant *psbA* gene is currently under weak selective constraints for translation efficiency, the other is that there are no current selective constraints and we are observing the remnants of an ancestral codon adaptation that is decaying under mutational pressure. We test these two models using simulations studies that incorporate the context-dependent mutational properties of plant chloroplast DNA. We first reconstruct ancestral sequences and then simulate their evolution in the absence of selection on codon usage by using mutation dynamics estimated from intergenic regions. The results show that *psbA* has a significantly higher level of codon adaptation than expected while other chloroplast genes are within the range predicted by the simulations. These results suggest that there have been selective constraints on the codon usage of the flowering plant *psbA* gene during Angiosperm evolution.

## Author summary

We simulated the evolution of four photosynthesis genes that are coded by the chloroplast genome of flowering plants in order to investigate the role of natural selection. In particular we were interested in whether or not selection can influence the evolution of features that do not affect the protein coded by the gene but that do affect the expression of the gene. We developed a model of mutation and then assume that there is no selection in simulations to generate expected patterns of evolution. Importantly, our mutation model accounts for a complex feature of chloroplast DNA which is that nucleotides near the site mutating affect the type of mutation that occurs. We found that one gene in particular has not evolved as predicted by our simulations but, rather, has evolved in a manner that suggests that mutations which affect the level of gene expression have been under natural selection.

## Introduction

Codon usage bias (CUB) results from an interplay between mutation dynamics and selection [1–3]. One well characterized selective pressure on codon usage is to increase translational efficiency, which includes increasing translation rate and possibly additional factors such as accuracy [2,3], resulting in a specific form of CUB called codon adaptation. This type of adaptation is characterized by a bias towards a specific set of codons, the so-called optimal or major codons. The strength of this selective pressure varies across genomes and also varies amongst genes within a genome, generally as a result of variation in expression level, with the result that in a limited set of highly expressed genes of certain genomes we see a strong bias towards major codons indicating a high level of codon adaptation [1,4–6]. Other selective pressures have also been proposed to influence codon usage, such as mRNA stability and protein folding [2], which are not mutually exclusive of codon adaptation, but the degree to which such factors influence codon usage is an open question.

Genes coded by the chloroplast genome provide an interesting example of CUB and codon adaptation. In some green algae, a prime example being *Chlamydomonas reinhardtii*, most genes have a CUB dominated by a strong A+T bias at synonymous positions, consistent with the genome composition bias. In contrast, a set of highly expressed genes, such as the core photosystem II protein coding gene *psbA* and the *rbcL* gene, which codes for the large subunit of RuBisCO, show strong evidence for codon adaptation [5–7]. These highly expressed genes are strongly biased towards a subset of codons that match the limited tRNA population in the chloroplast and that have base composition biases in certain synonymous codon groups that do not match the compositional bias of the genome overall [5–7]. For example, although the *C*. *reinhardtii* genome is roughly 35% G+C overall [8], 93.8% of the codons for the twofold degenerate amino acids Phe, His, Tyr, Cys, Asn, and Asp in the *psbA* gene have a C at the degenerate third position [6].

In Angiosperm chloroplast genomes there is little evidence for codon adaptation [5]. The CUB of almost every gene is dominated by a bias towards A+T which reflects the genome wide A+T bias. An interesting, and sole, exception is the *psbA* gene which codes for the predominant translation product of plant chloroplasts [5,6]. This gene uses certain codons at a slightly higher frequency than do other Angiosperm chloroplast genes and these are the same codons found at high frequency in highly expressed chloroplast genes in *C*. *reinhardtii* and some other algae [5,7]. The atypical codon usage, the similarity to plastid genes in algae, and the high translation level of *psbA* [9] suggests that this flowering plant gene is under selection for codon adaptation. However, the bias towards the adaptive–major—codons is much weaker than in highly expressed algal genes like the *psbA* gene from *C*. *reinhardtii* indicating that the selective pressure is relatively weak in comparison to other lineages.

The weaker bias towards major codons in the Angiosperm *psbA* gene could be due to weaker selective pressure and/or a decreased effective population size in flowering plants but another possibility is that the Angiosperm *psbA* gene is not currently under selection for codon usage. Rather, it is possible that we are observing the remnants of an ancestral bias that is slowly decaying under mutation pressure [10]. In this study we set out to examine this possibility by simulating the evolution of chloroplast genes in the absence of selective pressure on synonymous changes. By reconstructing putative ancestral sequences for different chloroplast genes and simulating how rapidly codon adaptation should decay in the absence of selection we can compare extant sequences to the simulated levels of adaptation.

To take this approach we need to account for the complex mutational dynamics of plant chloroplast DNA [11–13]. Previous studies have shown that mutations are strongly context-dependent meaning that they are influenced by the composition of the sites immediately flanking the mutation: both G+C content and pyrimidine-purine content of the neighboring bases are correlated with mutation dynamics [11,13,14]. Using noncoding DNA from closely related chloroplast sequences across a wide range of Angiosperm taxa we generated context-dependent mutation matrices and then use these to simulate the evolution of putative ancestral genes reconstructed from the sequence data of 165 Angiosperm taxa. We then compared the simulation results of four chloroplast genes to the level of codon adaptation in the extant genes, measured by the degree of bias towards the adaptive pattern observed in *C*. *reinhardtii*.

The results indicate that the level of codon adaptation in *psbA*, although much lower than in algae such as *C*. *reinhardtii*, is significantly greater in the extant sequences measured than what is expected from our simulations. The same result is not observed for the other chloroplast genes in our analysis, which show levels of codon adaptation in line with the simulated results. This suggests that the Angiosperm *psbA* gene has been constrained by some level of selection for codon adaptation. The data do not allow us to conclude that these selective constraints are acting currently but do indicate that constraints have existed during flowering plant evolution. At the same time, all genes show a broader variation in CUB than expected from our simulations raising the possibility that there are some weak selective pressures other than codon adaptation that effect CUB of all chloroplast genes.

## Results

### Substitution sount matrices

We generated alignments from 280 taxa triplets and scored 2,845,876 noncoding sites within a conserved flanking base context. Of these sites 16,651 (0.00585%) had an observed substitution between the ingroup taxa. This reduces the probability of multiple hits per site to essentially zero and so we assume that our substitution matrices accurately reflect the relative instantaneous mutation probabilities.

The 10 Substitution Count Matrices (once complementary matrices had been merged) show significant context-dependent dynamics as has been observed previously in chloroplast DNA [12–14]. The relative rates of transitions and transversions vary with flanking base A+T content with the result that Ts:Tv and overall substitution rate vary widely across contexts and the relative rates of GC->AT and AT->GC substitutions tends to decrease within increasing G+C context (Table 1). We determined the stationary base composition vector for each of the 10 Probability Matrices derived from the Substitution Count Matrices and there is wide variation in predicted G+C content across contexts (Table 1), with a positive correlation between the predicted equilibrium G+C content of a site and the flanking base G+C content of that site. The equilibrium T-A and C-G skews for the 10 Probability Matrices are quite different and in general skew is stronger when the flanking bases on one strand are both purines (or pyrimidines on the complement). Overall, the data show that there is significant heterogeneity in mutation dynamics between different flanking base contexts indicating that accurate simulations require context-dependent substitution models.

**Table 1:** Context-dependent properties of the MC2020 model.

Context^§^	G+C TS:TV^†^	A+T TS:TV^†^	Sub. Rate^††^	GC:AT Rate^††^	EQUIL. G+C^‡^	EQuil. C-G^‡‡^	EQUIL. T-A^‡‡^
**A_A/c(T_T)**	0.84	0.33	0.76	2.50	21.3	- 32.7	- 32.6
**A_T**	2.23	0.41	0.53	2.03	26.3	3.4	3.8
**T_A**	0.99	0.32	0.77	2.20	26.4	- 2.8	- 3.5
**A_C/C(G_T)**	1.95	1.16	0.47	2.30	30.0	- 0.27	- 14.4
**A_G/C(C_T)**	4.26	1.50	0.63	1.82	34.1	- 38.4	- 23.4
**C_A/C(T_G)**	1.72	0.71	0.78	2.01	32.1	25.3	- 21.2
**G_A/C(T_C)**	1.12	0.82	0.49	2.94	25.5	- 24.3	- 33.9
**C_C/C(G_G)**	3.07	2.80	0.66	1.57	40.8	40.6	16.0
**C_G**	3.06	1.24	1.01	1.08	46.4	- 6.1	11.0
**G_C**	1.41	2.89	0.61	1.95	36.6	5.2	15.1

§–C(N_N) refers to the complement of the matrix. In these cases the two complementary contexts were combined as described in the text

†—TS:TV at sites where the outgroup is a G/C or A/T

††–Substitutions/site x 100. GC:AT Rate gives the ratio of the rate of substitution from an ancestral G or C to the rate of substitution from an ancestral A or T

‡–Equilibrium G+C for the stationary vector of the matrix (see text)

‡‡–Equilibrium C-G and T-A skews for the stationary vector of the matrix

### Simulations

**Fig 1:**
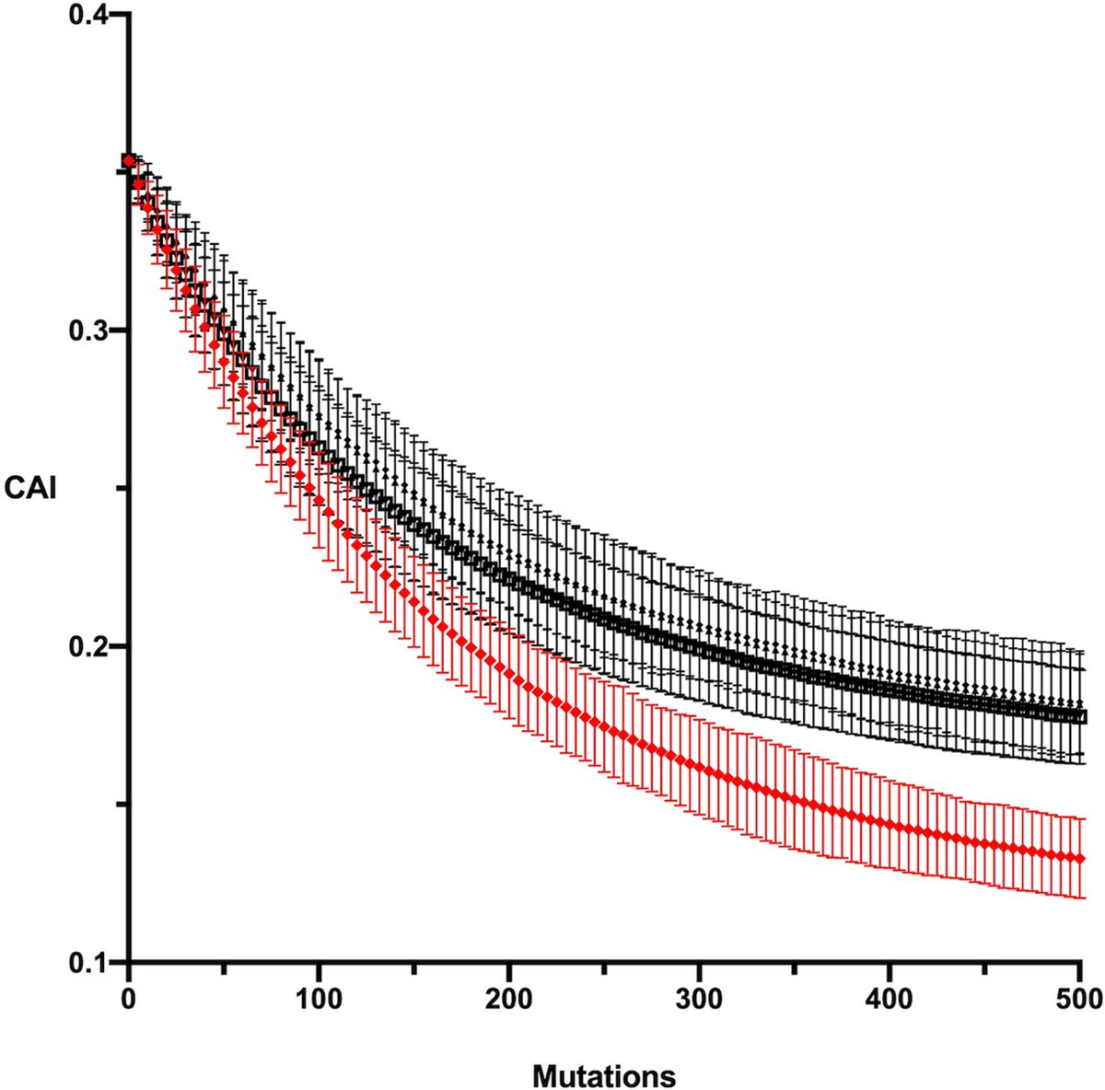
Simulation results for the ML_AA ancestral sequence of *psbA* under 5 models; MC with synonymous substitutions only, MC with nonsynonymous substitutions, MC2020 with synonymous substitutions only, MC2020 with nonsynonymous substitutions, and K2P with synonymous substitutions only. The four MC and MC2020 models are shown in black and the K2P model in red.

The three mutation models, K2P, MC and MC2020 as well as the different reconstructed ancestral sequences, ML-AA, ML-DNA, MP-High and MP-Low, are described in the Materials and Methods. The simulation results for the ML-AA ancestral *psbA* gene under each of the substitution models are shown in Fig 1. For the context-dependent models (MC and MC2020) both the synonymous and nonsynonymous simulations, the former restricted to synonymous changes and the latter taking into account amino acid changes as described in the Materials and Methods, are shown while for the K2P model only the synonymous simulation is shown. The decay in codon adaptation (measured by CAI) over time that is predicted by each model is taken as the predicted evolution of codon adaptation in the absence of selective constraints on codon usage. In each simulation we see a decline towards an equilibrium CAI value although the two context-dependent models, MC and MC2020, have a higher equilibrium CAI than does the K2P model. The deviation between K2P and the context-dependent models illustrates the importance of context dependency for the analysis. We will exclude K2P from hereon. Additionally, given the similarity between the results for the MC and MC2020 models we will only present data for the latter.

**Fig 2:**
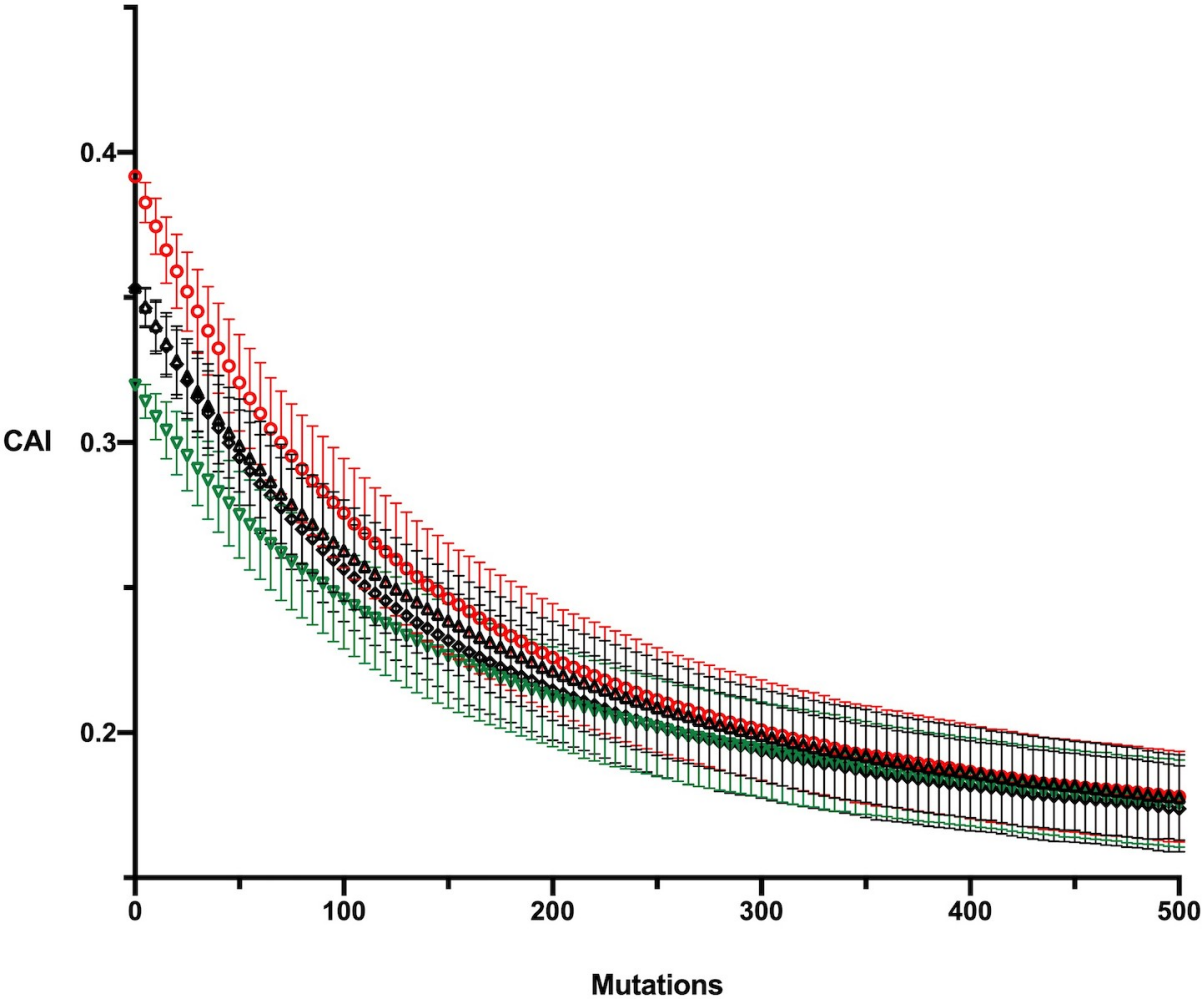
Simulation results for the MC2020 model with synonymous substitutions only for the four reconstructed ancestral sequences of *psbA*; MP-High (red), MP-Low (green) and ML-AA and ML-DNA (both black).

**Fig 3:**
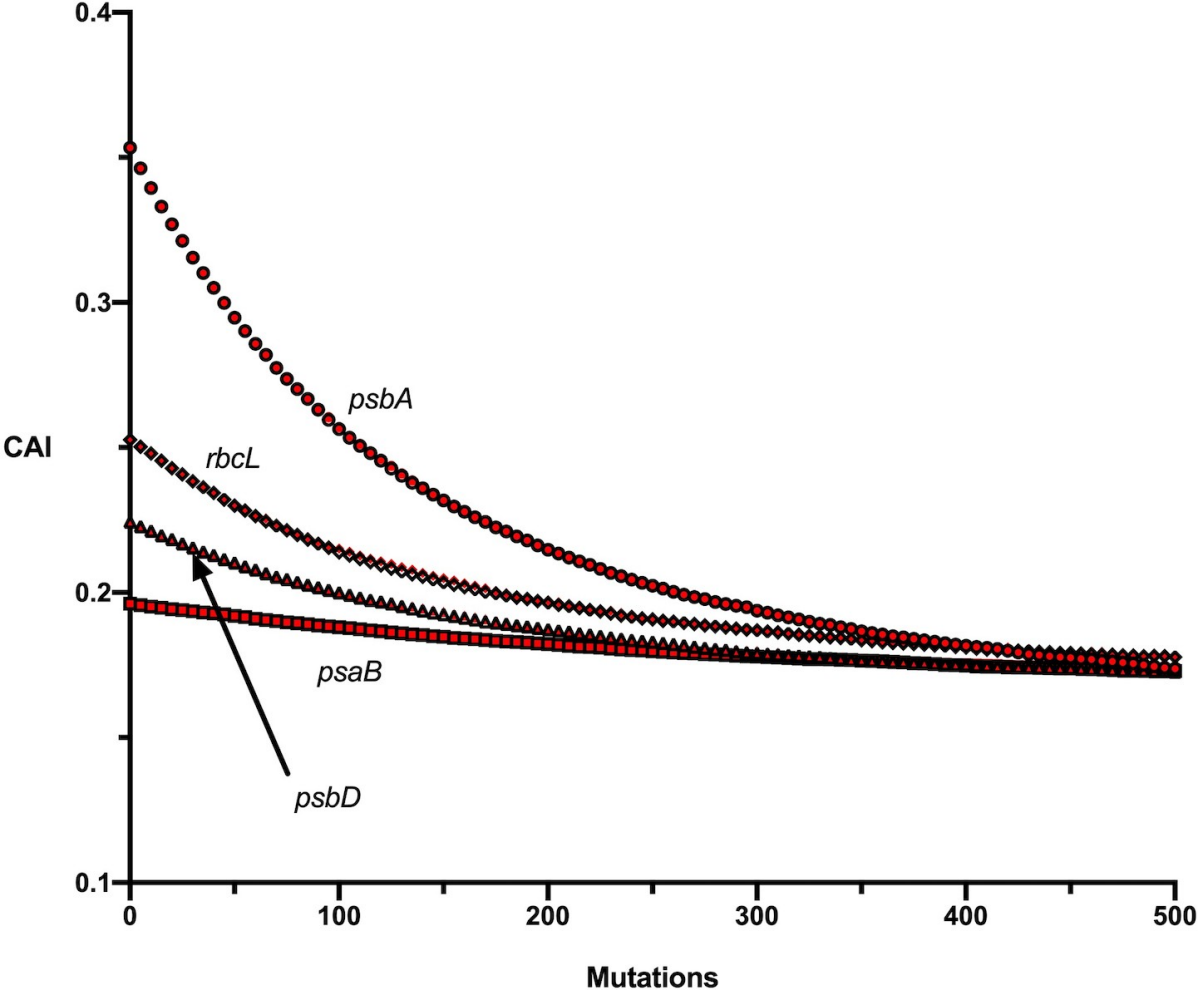
Simulation results for the ML-AA ancestor of each gene under the MC2020 model with synonymous substitutions only (black) and with nonsynonymous substitutions included (red). Standard deviations are not shown for clarity of presentation.

The simulation results for each of the four ancestral *psbA* sequences under the MC2020 model with only synonymous substitutions incorporated are shown Fig 2. The four ancestral sequences decay to essentially the same equilibrium codon adaptation level and we can take the area enclosed by the four curves as the area containing the predicted level of codon adaptation for *psbA* over time. Fig 3 shows the simulation results for the ML-DNA ancestral sequence of each gene under the MC2020 model. The simulations restricted to synonymous changes are virtually indistinguishable from those allowing nonsynonymous changes. Similar results were found for each method of ancestral reconstruction and for the MC substitution model indicating that the inclusion of acceptable nonsynonymous changes does not significantly alter the predicted evolution of codon adaptation. This similarity is likely due to the conserved nature of these proteins. For example, the monocot *Zea mays* (NC_001666) and dicot *Nicotiana tabacum* (NC_001879) *psbA* genes differ at only 6 of 353 amino acids (4 of which are in the last 8 amino acids) and the *d*_N_/*d*_S_ values were 0.029 for *psbA*, 0.101 for *psbD*, 0.074 *rbcL* and 0.039 for *psaB*.

One noticeable similarity across the genes is that the ancestral sequences are all predicted to decline in codon adaptation (Fig 3), although much less so in the case of *psaB*. This means that in each case they have a slightly higher CAI than the mutation models predict for the equilibrium sequence. It is also apparent that the simulations predict that all of the genes evolve to a similar equilibrium level of codon adaptation. This suggests that this is the predicted level of major codon representation in the absence of selection and that amino acid content of a protein does not significantly affect the degree of codon adaptation as measured by CAI.

**Fig 4:**
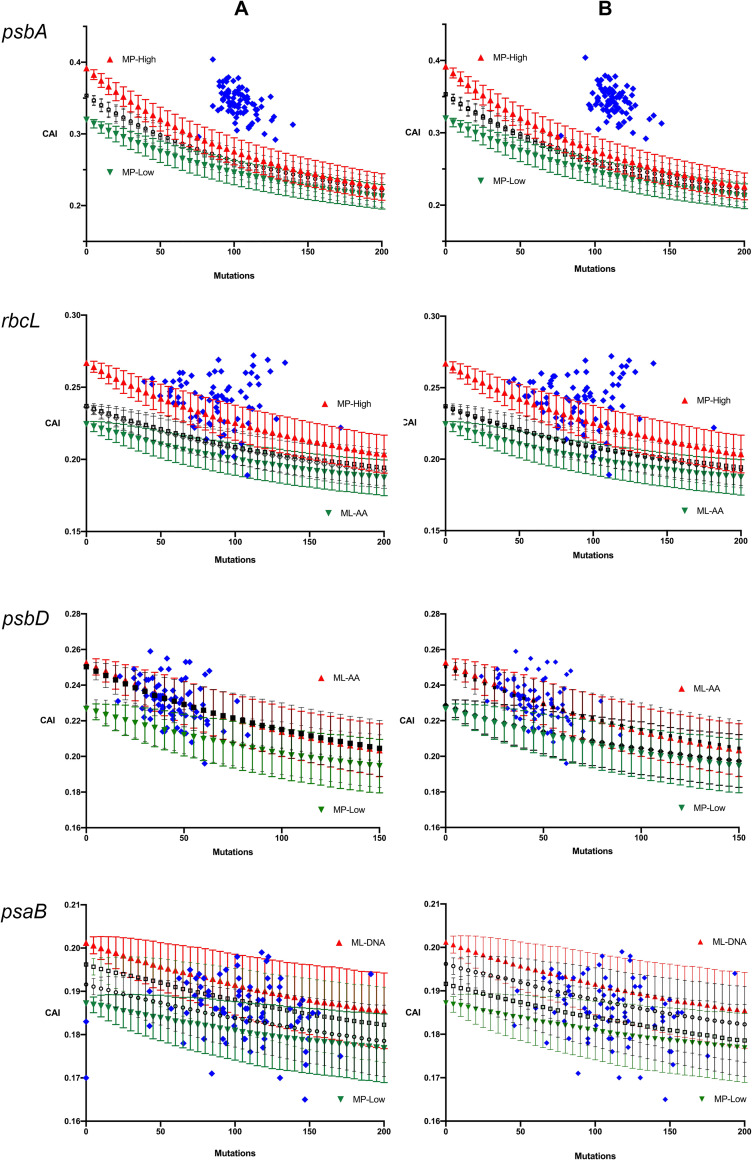
Comparisons of simulation results for each gene using the MC2020 model, with synonymous substitutions only, applied to each of the four ancestral sequences, with the extant sequence CAI values (blue). The ancestral sequences that yielded the highest and lowest simulation lines, determined by the CAI value of the ancestral sequences, are indicated and colored red and green respectively. For each gene the two plots are for the minimum (A) and maximum (B) number of mutations estimated from one of the reconstructed ancestral sequences (see text).

The simulation results using the MC2020 substitution model are compared to extant sequence CAI values for the four genes in Fig 4. For all genes the simulations are compared to extant sequences based on both the minimum and the maximum number of mutations between the ancestral and each extant sequence as described in the Materials and Methods. The variances for distance estimates were all very small and so they are not included in the plots for clarity purposes. The general results described below hold regardless of which mutation number is used as the observed number of mutations. In the case of *psbA* all extant genes have higher CAI values than predicted by the simulations except one which is within the standard deviation range of one simulation. These results suggest that the evolution of this gene is being, and/or has been, constrained by selection for codon adaptation during the divergence of the Angiosperms. For *rbcL* and *psbD* the extant genes have a range of CAI values spanning the interval predicted by the simulations although there is a trend towards more genes with higher CAI values than predicted. Given that the points are not independent this trend cannot easily be assessed. For *psaB* the extant genes have CAI values that span the predicted range. Overall, the results do not provide evidence for significant selective constraints on codon adaptation of *rbcL*, *psbD* or *psaB*.

## Discussion

This study aimed to gain an understanding of the selective pressure on the Angiosperm *psbA* gene, which has a codon usage pattern that is atypical for flowering plant chloroplast genes but which is similar to what is observed in a number of highly expressed algal chloroplast genes. Given the match between the chloroplast tRNA population and the turnover rate of the *psbA* protein product it had been proposed that the atypical pattern is the result of codon adaptation, or selection to increase the usage of major codons for translation efficiency [5,6]. An alternative hypothesis is that the extant *psbA* gene is not currently under selective constraints but may simply have the remnants of ancestral selection that is decaying towards a codon usage determined by the mutation bias in the chloroplast [10]. This alternative is suggested by the fact that although the codon usage pattern in Angiosperm *psbA* genes is similar to what is observed in highly expressed algal chloroplast genes, the level of adaptation, that is, the degree of bias towards major codons, is much lower in the Angiosperm *psbA* gene.

The data presented here suggest that the *psbA* gene has been under selective pressure during the evolutionary divergence of the Angiosperms although they are not conclusive about current selection. The simulation results indicate that the codon adaptation levels of extant Angiosperm *psbA* genes, measured by CAI using codon fitness values from *C*. *reinhardtii* chloroplast *psbA* and *rbcL* genes, are consistently greater than expected by our mutation models in the absence of selection on synonymous changes. Every mutation model and ancestral reconstruction predicts a more significant decay of codon adaptation over flowering plant evolution than what is observed. We ran simulations allowing only synonymous changes as well as simulations allowing amino acid changes that are observed in the alignment of Angiosperm sequences. Since the evolution of *psbA* is not consistent with either simulation we can conclude that selection at the amino acid level is not a factor in generating the results. In the other genes studied, we observe extant CAI values that overlap with all of the simulation predictions. Therefore, the evidence for selective constraints on codon adaptation is limited to *psbA*, the predominant translation product of the plant chloroplast [5,6,9]. Although the level of adaptation is much lower in Angiosperms than in algal chloroplast genes it does not appear to be the case that the *psbA* gene has been decaying towards a codon usage that is determined solely by mutational biases.

The choice of reference genes used to estimate codon fitness values could potentially affect the inferences drawn here. Running the *psbA* simulations with other highly expresses *Chlamydomonas* chloroplast genes as a reference set and, separately, with *rbcL* and *psbA* from the liverwort *Marchantia polymorpha* as a reference set did not affect the general finding. Since the set of major codons is shared across plastids [5] it is not surprising that any reference set of plastid genes under codon adaptation would yield the same general finding concerning the *psbA* gene from flowering plants.

Another important factor is the underlying mutation model employed in the simulation. Given the complex nature of mutation in chloroplasts [11,12,14] we elected to employ a context-dependent model derived from substitutions observed in intergenic regions of very closely related chloroplast genomes. The large number of substitutions used to estimate the mutation parameters and the fact that they are from highly similar sequences, which reduces the probability of multiple hits to essentially zero, makes it likely that this is the best representation of mutation dynamics, in terms of the relative probability of each type of mutation, that we could get for this type of analysis. However, our inference also requires the assumption that mutation models derived from intergenic regions are applicable to coding regions. Although we expect selection to act differently on the two sequence types, we assume that the underlying mutation probabilities are the same. Given the similarity between substitutions in intergenic regions and at fourfold degenerate sites in coding regions [14,15] this seems to be a valid assumption. Further, if there are factors that affect mutation dynamics in coding regions differently than in intergenic regions then we would expect them to be random with respect to any influence on the level of codon adaptation. Therefore, they would not explain the difference between *psbA* and the other chloroplast genes in terms of a retention of codon adaptation over Angiosperm evolution.

There are two features of the results that deserve further consideration. One is that all four genes show predicted decay in CAI across the simulations. This leads to the inference that all putative ancestral sequences have (or had) slightly higher codon adaptation than the equilibrium level predicted by the mutational model. Any significance of this is uncertain. One possibility is that mutation dynamics have evolved over time such that the equilibrium itself has changed. The difference between predicted equilibrium and ancestral CAI is very small, between about 0.01 and 0.05 for the genes in this study, but it would be informative to study the evolution of context-dependent mutation dynamics in chloroplast DNA. It could also be that there is, in fact, a small difference between codon and noncoding mutation dynamics, perhaps due to larger context effects, meaning an influence of nucleotides beyond the two neighboring bases, that accounts for this small difference. This would not explain, however, the difference between *psbA* and the other genes in terms of maintenance of codon adaptation across time.

The second notable feature is the unpredicted variation in CAI values of the extant genes. For each of the four genes the individual simulations predict a range of CAI values across time that is narrower than what we observe for any gene, regardless of the degree of overlap with the predicted range (see Fig 4). There are two main possibilities for this. One is the possibility discussed above, that although our MC and MC2020 models are based on a very large number of substitutions observed in noncoding regions and account for the context effects of the two flanking nucleotides, there may still be significant variation in mutational dynamics across sites that is not captured by our models. Another possibility is that there are actually selective constraints on silent changes in these genes that are not related to codon adaptation. In particular, it is possible that regulatory sequences or secondary structures impose selective constraints at specific sites that affects the evolution of the gene, but that are random with respect to CAI, leading to a greater dispersion of CAI values than expected. This scenario could be compounded by lineage-specific effects of any compensatory changes. As changes accumulate along one lineage they could alter selective pressures at other sites, for example due to compensatory changes observed at protein binding sites or secondary structures, leading to further dispersion in CAI across independently evolving lineages. This would be similar to the model for sequence divergence despite functional conservation at enhancer sequences [16,17]. The existence of selective pressures based on regulation or other factors not related to codon adaptation should be examined in future studies.

## Materials and methods

### Mutation models

Since it is known that flanking base composition has a strong influence on mutation dynamics in chloroplast DNA [11,13,14] we used observed substitutions in noncoding regions to develop two context-dependent models. One of these, called here the MC model, is the context-dependent model described previously [11]. The other, called here the MC2020 model, is a new context-dependent model generated from current NCBI data. As described below, we developed this model by counting the number of each substitution type across multiple genome alignments to yield context-dependent Substitution Count Matrices, and then used these to generate Probability Matrices with the relative probabilities of each type of mutation. For comparison we also used the Kimura 2 Parameter model [18], called here K2P, with a transition:transversion (Ts:Tv) ratio of 3:1.

For the MC2020 model, RefSeq complete chloroplast genome sequences were downloaded from NCBI (www.ncbi.nlm.nih.gov/genome/browse#!/eukaryotes/) on March 14 2019. All genomes annotated as Magnoliophyta were then parsed using the Biopython 1.76 [19] GenBank parser and sequences between neighboring Biopython SeqRecords extracted as intergenic regions. Genomes were grouped into closely related triplets using the following protocol and using the annotated taxon information in the NCBI files. All genomes from each Family were pooled and for any genus with at least two representative genomes, two were selected at random and paired. An outgroup taxon was then selected at random from the other genomes in that Family. From all remaining genomes in the family two were paired randomly and the rest discarded. This process was followed both to prevent duplicate sampling of evolutionary branches and, by selecting closely related genomes, to minimize multiple changes at a site along any branch so that the substitutions scored as described next can be used to infer the relative probability of each mutation.

### Substitution count matrices for model MC2020

Intergenic regions were aligned using the Clustal alignment function in Biopython with a gap open penalty of 2 and a gap extend penalty of 0.5. Substitution Count Matrices were generated from the intergenic alignments of regions greater than 70 nucleotides in length using a Python script written by BRM. A separate 4x4 count matrix was generated for each possible flanking base context, where context is defined as the base composition of the immediate 5’ and 3’ neighbors and will be referred to as N_N for the 5’ and 3’ neighbor respectively (e.g. the middle nucleotide in the sequence TGA is in the T_A context). In each context-dependent Substitution Count Matrix M, element M_ij_ is the number of sites at which an i → j substitution was observed, with diagonal elements M_ii_ counting sites at which nucleotide i is conserved. To generate the Substitution Count Matrices, all sites in the alignment were scored and any site with a conserved context in all 3 sequences was retained, those without a conserved context were ignored. Sites that were conserved between the ingroup pair were counted as a conserved site (e.g. G → G) while those that had a substitution differentiating the ingroup sequences were counted as having one N_a_ → N_d_ substitution, where N_a_ is the ancestral nucleotide and N_d_ the derived. The ancestral state was inferred from the outgroup sequence if it was the same as one of the ingroup sequences. If all 3 sequences differed the site was ignored.

Once the 16 Substitution Count Matrices were generated, complementary matrices were combined. For example, the A_A context and the T_T context count matrices were combined by adding the complementary count matrix of one to the other count matrix. The combined A_A count matrix is the A_A matrix added to the complementary T_T matrix while the T_T count matrix is then just the complement of this (which can be generated from the original T_T count matrix combined to the complement of the original A_A count matrix). This yielded a total of 10 different context-dependent Substitution Count Matrices that can be used for all 16 contexts in a strand-specific analysis.

### Probability matrices

For the MC2020 model, each context-dependent Substitution Count Matrix was converted to a Probability Matrix P, in which P_ij_ is the relative probability of change from base i to base j given a that there is a mutation of base i. Each context-dependent matrix P was calculated by dividing each element in the relevant (i.e. same context) Substitution Count Matrix by the row total. In P each row will sum to one and the diagonal elements, representing a mutation from i to i, are non-zero. These ‘non-mutations’ are included to simulate variation in the overall mutation rate from different bases (e.g. a higher rate of mutation of Gs than of Ts). This is described in the Simulations section below but an important point is that the manner in which we simulate this slows the simulation run time. Therefore, we normalized all rates in order to minimize run time while retaining relative rates across all matrices. First, across all Probability Matrices the row with the highest overall rate, which is the sum of the non-diagonal elements in the row, was determined. We then rescaled each Probability Matrix such that the overall rate in each row was the same fraction of the maximum rate across all matrices while preserving the relative rates within each row. Model MC was used as described previously [11] and for the K2P model we generated a Probability Matrix with a 3:1 Ts:Tv ratio.

These Probability Matrices are also Markov Process Transition Matrices and so for comparative purposes the stationary vector π was determined for each matrix, such that πP = π, by calculating P^t^ for increasing t until the rows reach equality at which point each row will be equal to π [20]. This vector is equal to the equilibrium base frequency for a sequence evolving with the mutation dynamics given by the matrix.

### Codon fitness and Codon Adaptation

Codon usage was measured using the Codon Adaptation Index (CAI) of Sharp and Li [21]. This is a measure of adaptation based on codon fitness values estimated from genes inferred to be under strong selection for a particular codon usage pattern. Based on previous work [5,6] we chose *psbA* and *rbcL* from *Chlamydomonas reinhardtii* as reference genes to estimate codon fitness values. These genes show evidence for strong adaptation for translational efficiency and the codon usage they display is the same as the pattern favored by selection for translation efficiency in Angiosperm chloroplasts as indicated by the *psbA* codon usage [5,6,9] although they show much stronger adaptation than any Angiosperm chloroplast gene. The resulting CAI for any gene is a measure of the degree to which the gene uses high fitness codons (the major codons) as estimated from these reference genes.

### Phylogenetic and ancestral sequence reconstruction

We selected 165 Angiosperm taxa (S1 Table) and 4 outgroup taxa to use for phylogenetic analysis and ancestral reconstruction. The 165 taxa were selected to provide a broad coverage of flowering plant lineages. From each Order represented in the NCBI RefSeq chloroplast genome dataset we selected at random a taxon from each of the Families represented. For the outgroup taxa we selected 4 RefSeq genomes; the Gymnosperms *Pinus thunbergii* and *Welwitschia mirabilis*, the cycad *Zamia furfuracea*, and the Ginkgophyta *Ginkgo biloba*.

For each gene four different ancestral sequences were reconstructed, two using Maximum Likelihood and two using Maximum Parsimony. First, the 169 *psbA* amino acid sequences were aligned using default parameters in Clustal Omega [22] and then a Maximum Likelihood phylogeny with PhyML [23]. For each of the genes *psbA*, *rbcL*, *psbD* and *psaB*, we then aligned the DNA sequences from the same 169 taxa using Clustal Omega. The topology of the *psbA* protein tree was then used for a parsimony inference of the ancestral Angiosperm DNA sequence for each gene using Mesquite [24]. Sites at which there was an ambiguity were resolved in two separate ways based on the codon fitness values as described above. One ancestral sequence had all ambiguities resolved to the base at each site that yielded the synonymous codon with the lowest fitness, while the second was resolved at each site to the synonymous codon with the highest fitness value. These two ancestral sequences, called MP-Low and MP-High are taken as representing the outside extremes of ancestral codon adaptation for the gene as inferred by parsimony. Using the same tree topology we then used IQTree [25,26] to reconstruct the maximum likelihood ancestral sequence which we will refer to as ML-AA.

For each gene we also aligned the 169 DNA sequences using Clustal Omega and then reconstructed the ML phylogeny using the GTR model in IQTree with the FreeRate variation model. The ML sequence in the IQTree output at the node representing the Angiosperm ancestor was taken as the fourth ancestral sequence which we will refer to as ML-DNA.

### Simulations

The Probability Matrices were used to simulate the evolution of *psbA*, *rbcL*, *psbD* and *psaB*. The basic simulation process followed two steps, the first incorporating the mutation model and the second incorporating selection. In the first step, a site was chosen at random across the length of the gene and the appropriate context-dependent Probability Matrix used to select a potential mutation at random. The second step, selection, involved deciding whether or not the potential mutation became a mutation to be introduced into the sequence. This involved selecting out potential mutations that introduced unacceptable amino acid replacements as described below, retaining only ‘acceptable’ replacements. Along with context dependency and selection, the simulation process accounts for two important factors. One is the variation in context, and thus mutational dynamics, of a site over time as flanking bases are mutated and the other, as a result of non-zero diagonal values in the Probability Matrices, is the variation in overall mutation rate across contexts as well as variation in the mutation rate of different nucleotides within the same context.

For each gene we ran 24 simulations, 6 from each of the four inferred ancestral sequences. The 6 simulations consisted of two simulations for each of the three mutation models. One set of simulations introduced only synonymous changes, the second allowed nonsynonymous changes based on the following method. For each gene we generated a list of observed amino acid replacements at each site in the 165 Angiosperms taxa described above. These were taken as ‘acceptable’ replacements at that site. We also calculated the *d*_N_/*d*_S_ ratio for each gene using PAMLX [27], which was taken as the probability of a nonsynonymous relative to a synonymous substitution. The acceptable changes and the *d*_N_/*d*_S_ ratio were used as limiting factors in the simulations as described below.

Simulations were run using Python code generated in-house on the Spyder IDE (www.spyder-ide.org/). Each simulation involved introducing 500 mutations into a specified ancestral DNA sequence and we ran 1000 replicates for each simulation. The CAI value was calculated after every 5 mutations and the average and standard deviation of the 1000 replicates calculated for this point. Each round of a simulation involved two steps as described above. In the first step, a potential mutation was generated. A site was chosen along the length of the gene using a uniformly distributed random variable. The flanking bases of the site chosen were used to select the appropriate context-dependent Probability Matrix and the existing nucleotide at the site determined the row in the matrix. The new nucleotide, which is the potential mutation, was selected at random based on the relative probabilities in that Probability Matrix row. In the second step, the potential mutation was either accepted–becoming a mutation incorporated into the sequence—or discarded as follows. The first criterion was that the potential mutation was to a novel base. Since the Probability Matrices have non-zero diagonals it was possible for the new nucleotide to be the same as the original. In this case the potential mutation was discarded since it is not in fact a mutation. This allows us to capture rate variation across sites as a function of flanking base content as well as variation between the mutation rates of the four nucleotides within each context. The second criterion was based on the amino acid sequence coded. For the simulations allowing nonsynonymous changes it was first determined whether or not it was an acceptable amino acid replacement as described above. If not, the potential mutation was discarded. If the replacement was acceptable it was accepted, becoming a mutation incorporated into the sequence, with a probability equal to *d*_N_/*d*_S_ of that gene, otherwise it was discarded. All synonymous potential mutations were accepted. In the simulations restricted to synonymous changes any nonsynonymous change was discarded and all synonymous mutations were accepted. Whenever a potential mutation was discarded the program returned to the site selection stage. This process was repeated until 500 mutations had been incorporated into the sequence. All alignments, ancestral sequences and code are available at Dryad [28].

### Codon adaptation of extant genes

For 81 of the 165 Angiosperm taxa, chosen to represent all Orders in our dataset, we calculated CAI values for *psbA*, *rbcL*, *psbD* and *psaB* as described above. To estimate the number of substitutions separating the ancestral sequence and each extant sequence we aligned each gene from each taxon to each of the reconstructed ancestral sequences using the needle2 program from EMBOSS [22]. From the alignments we calculated a k value and variance using the K2P model, which was used because of the very low number of differences between the sequences. The k value was multiplied by the length of the gene to generate the inferred number of mutations. Therefore, each gene had two distance estimates, a minimum and a maximum, for comparison to the simulation results.

## Supporting information

S1 Table List of taxa used in the substitution count analysis. In all cases the RefSeq genome file was downloaded from https://www.ncbi.nlm.nih.gov/genome/browse#!/eukaryotes/.
(DOCX)
