# Supplementary material for: Evidence from simulation studies for selective constraints on the codon usage of the Angiosperm psbA gene
Source: PLoS Comput Biol. 2021 Oct 26;17(10):e1009535. doi: 10.1371/journal.pcbi.1009535 (PMC8570520; doi:10.1371/journal.pcbi.1009535)
Supplement: S1 Table — In all cases the RefSeq genome file was downloaded from https://www.ncbi.nlm.nih.gov/genome/browse#!/eukaryotes/. (DOCX) [file pcbi.1009535.s001.docx]

**S1 Table**. List of taxa used in the substitution count analysis. In all cases the RefSeq genome file was downloaded from <https://www.ncbi.nlm.nih.gov/genome/browse#!/eukaryotes/>

| \| *Acorus gramineus* \| \| --- \| \| *Actinidia kolomikta* \| \| *Adoxa moschatellina* \| \| *Akebia quintata* \| \| *Aletris spicata* \| \| *Allium cepa* \| \| *Aloe maculata* \| \| *Aloysia citrodora* \| \| *Amaranthus hypochondriacus* \| \| *Ananas comosus* \| \| *Anemopaegma chamberlaynii* \| \| *Annona cherimola* \| \| *Aquilaria sinensis* \| \| *Aralia undulata* \| \| *Areca vestiaria* \| \| *Asarum minus* \| \| *Asclepias syriaca* \| \| *Averrhoa carambola* \| \| *Barringtonia racemosa* \| \| *Berberis bealei* \| \| *Betula cordifolia* \| \| *Boehmeria umbrosa* \| \| *Brassica juncea* \| \| *Burmannia oblonga* \| \| *Buxus microphylla* \| \| *Byrsonima crassifolia* \| \| *Cabomba caroliniana* \| \| *Calycanthus floridus* \| \| *Camellia cuspidata* \| \| *Campanula punctata* \| \| *Campynema lineare* \| \| *Carapa guianensis* \| \| *Carex neurocarpa* \| \| *Carica papaya* \| \| *Carludovica palmata* \| \| *Carnegiea gigantea* \| \| *Carpodetus serratus* \| \| *Ceratophyllum demersum* \| \| *Champereia manillana* \| \| *Chloranthus japonicus* \| \| *Chrysanthemum indicum* \| \| *Chrysobalanus icaco* \| \| *Circaeaster agrestis* \| \| *Cistantha longiscapa* \| \| *Citrus platymamma* \| \| *Coffea canephora* \| \| *Colchicum autumnale* \| \| *Commiphora wightii* \| \| *Cornus controversa* \| \| *Corynocarpus laevigatus* \| \| *Cucurbita maxima* \| \| *Cuscuta reflexa* \| \| *Dasypogon bromeliifolius* \| \| *Davidia involucrata* \| \| *Dioscorea villosa* \| \| *Diospyros blancoi* \| \| *Drimys granadensis* \| \| *Drosera regia* \| \| *Echinacanthus lofouensis* \| \| *Elaeagnus mollis* \| \| *Erythropalum scandens* \| \| *Erythroxylum novogranatense* \| \| *Eucalyptus torquata* \| \| *Euonymus hamiltonianus* \| \| *Euphorbia esula* \| \| *Euptelea pleiosperma* \| \| *Fagus engleriana* \| \| *Francoa sonchifolia* \| \| *Garcinia mangostana* \| \| *Genlisea filiformis* \| \| *Gentiana straminea* \| \| *Geranium palmatum*  *Ginkgo biloba* \| \| *Habenaria pantlingiana* \| \| *Hanguana malayana* \| \| *Heliconia collinsiana* \| \| *Helwingia himalaica* \| \| *Hibiscus syriacus* \| \| *Humulus lupulus* \| \| *Hydrangea luteovenosa* \| \| *Illicium oligandrum* \| \| *Impatiens piufanensis* \| \| *Iodes cirrhosa* \| \| *Iris sanguinea* \| \| *Japonolirion osense* \| \| *Joinvillea ascendens* \| \| *Juglans regia* \| \| *Lagerstroemia speciosa* \| \| *Lamium galeobdolon* \| \| *Lancea tibetica* \| \| *Larrea tridentata* \| \| *Leitneria floridana* \| \| *Lilium fargesii* \| \| *Linum usitatissimum* \| \| *Liquidambar formasana* \| \| *Lonicera tragophylla* \| \| *Luzuriaga radicans* \| \| *Macadamia ternifolia* \| \| *Magnolia biondii* \| \| *Melastoma candidum* \| \| *Morella rubra* \| \| *Morus mongolica* \| \| *Musa itinerans* \| \| *Najas flexilis* \| \| *Nelumbo nucifera* \| \| *Nymphaea mexicana* \| \| *Oenothera oakesiana* \| \| *Olea woodiana* \| \| *Ostericum grosseserratum* \| \| *Oxyria sinensis* \| \| *Paeonia obovata* \| \| *Papaver somniferum* \| \| *Paris quadrifolia* \| \| *Parrotia subaequalis* \| \| *Passiflora edulis* \| \| *Paulownia tomentosa* \| \| *Pentaphylax euryoides* \| \| *Penthorum chinense* \| \| *Phoebe zhennan*  *Pinus thunbergii* \| \| *Piper kadsura* \| \| *Plantago media* \| \| *Platanus occidentalis* \| \| *Portulaca oleracea* \| \| *Potamogeton perfoliatus* \| \| *Pouteria campechiana* \| \| *Primula poissonii* \| \| *Primulina liboensis* \| \| *Ranunculus macranthus* \| \| *Ravenala madagascariensis* \| \| *Rosa roxburghii* \| \| *Sabia yunnanensis* \| \| *Sagittaria lichuanensis* \| \| *Salix magnifica* \| \| *Sapindus mukorossi* \| \| *Schoepfia jasminodora* \| \| *Scrophularia henryi* \| \| *Sedum sarmentosum* \| \| *Sesamum indicum* \| \| *Silene chalcedonica* \| \| *Sladenia celastrifolia* \| \| *Solanum cheesmaniae* \| \| *Stephanis japonica* \| \| *Styrax grandiflorus* \| \| *Symplocarpus renifolius* \| \| *Symplocos ovatilobata* \| \| *Tapiscia sinensis* \| \| *Tarenaya hassleriana* \| \| *Taxillus sutchuenensis* \| \| *Tetragonia tetragonioides* \| \| *Tofieldia thibetica* \| \| *Torricellia angulata* \| \| *Trithuria inconspicua* \| \| *Trochodendron aralioides* \| \| *Typha latifolia* \| \| *Ulmus macrocarpa* \| \| *Vaccinium macrocarpon* \| \| *Viola seoulensis* \| \| *Viscum coloratum* \| \| *Vitis mustangensis* \| \| *Welwitschia mirabilis* \| \| *Wisteria floribunda* \| \| *Yucca filamentosa* \| \| *Zamia furfuracea* \| \| *Zea luxurians* \| \| *Zingiber spectabile* \| \| *Ziziphus jujuba* \| \| *Zostera marina* \| |  |
| --- | --- | --- | --- | --- | --- | --- | --- | --- | --- | --- | --- | --- | --- | --- | --- | --- | --- | --- | --- | --- | --- | --- | --- | --- | --- | --- | --- | --- | --- | --- | --- | --- | --- | --- | --- | --- | --- | --- | --- | --- | --- | --- | --- | --- | --- | --- | --- | --- | --- | --- | --- | --- | --- | --- | --- | --- | --- | --- | --- | --- | --- | --- | --- | --- | --- | --- | --- | --- | --- | --- | --- | --- | --- | --- | --- | --- | --- | --- | --- | --- | --- | --- | --- | --- | --- | --- | --- | --- | --- | --- | --- | --- | --- | --- | --- | --- | --- | --- | --- | --- | --- | --- | --- | --- | --- | --- | --- | --- | --- | --- | --- | --- | --- | --- | --- | --- | --- | --- | --- | --- | --- | --- | --- | --- | --- | --- | --- | --- | --- | --- | --- | --- | --- | --- | --- | --- | --- | --- | --- | --- | --- | --- | --- | --- | --- | --- | --- | --- | --- | --- | --- | --- | --- | --- | --- | --- | --- | --- | --- | --- | --- | --- | --- | --- | --- | --- | --- |
